# Lung adenocarcinoma associated with cystic airspaces

**DOI:** 10.1002/cdt3.51

**Published:** 2022-11-17

**Authors:** Xue Yang, Linlin Zhang, Fanlu Meng, Wenjing Song, Dong Li, Diansheng Zhong

**Affiliations:** ^1^ Department of Medical Oncology Tianjin Medical University General Hospital Tianjin China; ^2^ Department of Pathology Tianjin Medical University Tianjin China; ^3^ Department of Pathology Tianjin Medical University General Hospital Tianjin China; ^4^ Department of Radiology Tianjin Medical University General Hospital Tianjin China

To the Editor,

Lung cancer associated with cystic airspaces is a group of uncommon lung malignant lesions that are easy to misdiagnose even though the use of computed tomography (CT) has been more common for lung cancer screening in the clinic. The incidence of this lesion has been reported at 0.5%–3.7% in different studies.^1^, ^2^, ^3^ Up to 80% of patients was of pulmonary adenocarcinomas and most patients had a history of smoking.^2^

Here, we reported a case of lung adenocarcinoma associated with cystic airspace developed at least 7 years progression from one purely thin‐walled lung cavity closing to the pleura on CT images in a 53‐year‐old man without a smoking history. Histopathology revealed the thickened wall of the cystic airspace was lung adenocarcinoma with extensive papillary and micropapillary patterns and psammoma bodies.

A 53‐year‐old asymptomatic male without a smoking history has had a physical examination every 1–2 years since 2013. The patient has no history of asbestos and dust exposure. In December 2013, a local thin‐wall air cavity in the right lower lobe close to the pleura (Figure 1A) and nodular thickening of the bilateral pleura with partial calcification (data not shown) were found for the first time on his CT scan. Five months later, this patient underwent a chest CT examination again and there was no obvious change compared to the CT scan in 2013 (Figure 1B). After that, he did a chest CT examination every 1–2 years. No obvious radiological change was found (Figure 1C,D). However, in Oct 2018, a nonsolid nodule was found extruding from the local wall of the cystic airspace (Figure 1E), but these changes did not attract the attention of radiologists and physicians. Two years later, this nodule increased in size, and the wall of the cystic airspace thickened, which was considered the manifestation of lung cancer (Figure 1F). From 2013 to 2020, this patient had no discomfort.

**Figure 1:**
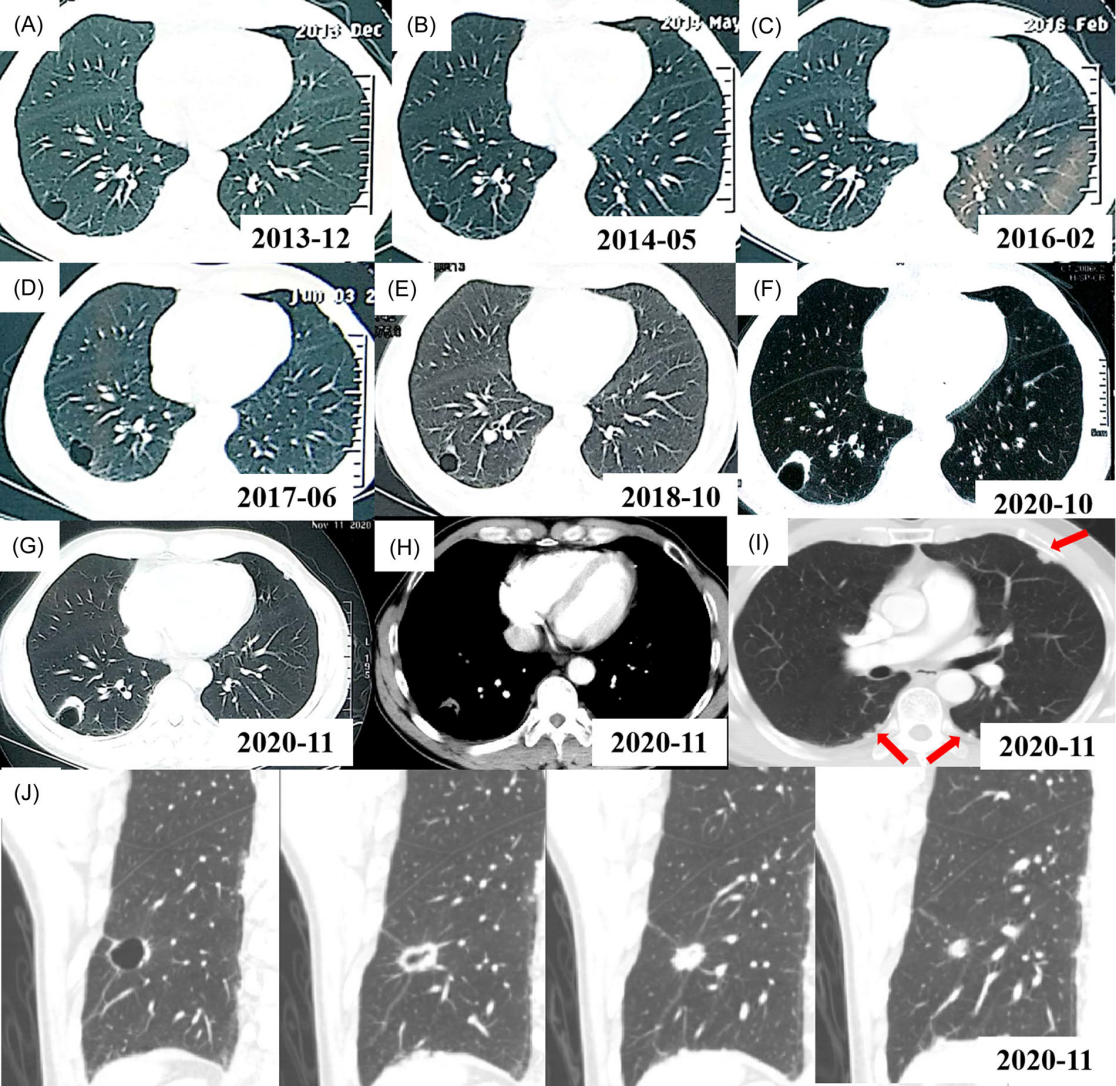
Computed tomography (CT) scans of the lung cystic airspaces from 2013 to 2020. (A) Initial CT shows a local thin‐walled air cavity in the right lower lobe in Dec 2013. (B–D) On a CT follow‐up, the size of the thin‐walled air cavity changed little in May 2014 (B), Feb 2016 (C), and Jun 2017 (D), respectively. (E) CT shows that a nonsolid nodule appeared adjacent to the wall of cystic airspace in Oct 2018. (F) CT shows that the size of the nodule increased and the wall of the cystic airspace thicken in Oct 2020. (G) CT shows a thicken‐walled cystic space with exophytic solid nodule along the cyst wall in Nov 2020. (H) CT shows a soft tissue nodule in the mediastinal window in Nov 2020. (I) CT shows local pleura irregular thickening and multiple small nodules on both lungs in Nov 2020. (J) CT images of the lung cystic airspace and nodule after three‐dimensional reconstruction in November 2020.

In December 2020, he was administrated to our hospital for further diagnosis and treatment. A physical examination of this case showed no obvious abnormality. Axial CT images in a lung window setting showed a thicken‐walled cystic space with exophytic solid nodules along the cyst wall (Figure 1G). A soft tissue nodule was seen in the mediastinal window (Figure 1H). An irregular thick‐walled cavity with local solid nodule formation after three‐dimensional reconstruction can be easily detected (Figure 1I). Bilateral pleurae showed irregular thickening and multiple small nodules and calcifications (Figure 1J).

After exclusion of extrathoracic malignancies by examination of brain CT scan and single‐photon emission computed tomography (SPECT) whole bone scan, resection of the cyst with nodules was performed and the malignant tumor was reported via intraoperative frozen tissue pathology. So, a right lower lobectomy with lymph node dissection was subsequently performed. Postoperative pathology confirmed that there were no metastases in the margins, broken ends of the bronchus, pleural, and peripheral lymph nodes (2–4 group [0/1], 7 group [0/8], 8 group [0/1], 11 group [0/4]). The resection specimen showed that the size of the cyst is about 2.0 × 1.5 × 1.0 cm. Histopathology examination revealed the nodule extruding from the local wall of the cystic airspace was pulmonary adenocarcinoma with extensive papillary and micropapillary patterns and psammoma bodies (Figure 2A). Hematoxylin and eosin (HE) staining of tumor specimen suggested that there was no defined lining between the tumor tissue and the cavity (Figure 2A). Immunohistochemical staining showed the tumor cells were strongly positive for thyroid transcription factor 1 (TTF‐1) (Figure 2B) and NapsinA (Figure 2C), positive for cytokeratin (CK) 19, Galectin 3, Cyclin D1, carcino‐embryonic antigen (CEA) and CK7 (data not shown), while negative for Tg (Figure 2D), CDX2, P40, CK5/6 and CK20 (data not shown). The ki‐67 index was about 20% revealing slower cell proliferation (data not shown). The tumor cells were also negative for programmed cell death ligand 1 (PD‐L1) expression with tumor cell proportion score (TPS) < 1% and combined positive score (CPS) = 10 (Figure 2E). The size of one resected grayish‐yellow pleural nodule was about 0.6 cm × 0.4 cm in size and was a pure calcific nodule without metastasis of pulmonary adenocarcinoma by HE staining (Figure 2F).

**Figure 2:**
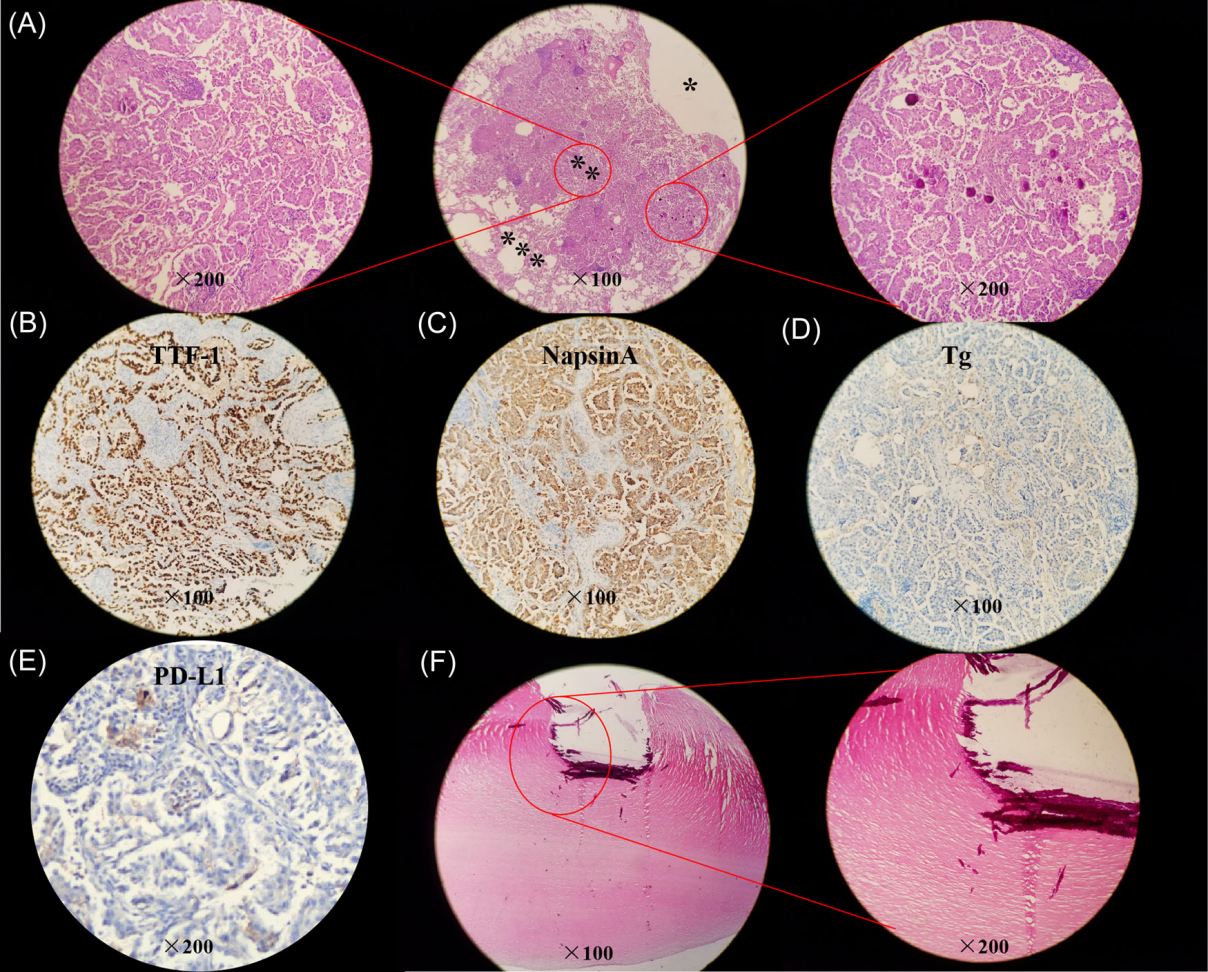
Histopathology and immumohistochemical staining of main makers. (A) Hematoxylin and eosin (HE) staining of resected lung specimen (*lung cavity; **tumor issue; ***normal lung tissue). (B–E) Tumor cells are positive for TTF‐1 (B) and NapsinA (C), while negative for Tg (D) and PD‐L1 (E, Dako 22C3 antibody). (F) HE staining of the resected pleural nodule.

A lung adenocarcinoma staged at pT1cN0M0 (Stage IA3) was diagnosed for this case. Epidermal growth factor receptor (EGFR) mutations and anaplastic lymphoma kinase (ALK) rearrangements were not found in the tumor. The tumor mutation burden (TMB) was low with 3.91 Muts/Mb. The state of microsatellite was microsatellite stable (MSS). There is no tumor recurrence until Apr 2022 (Supporting Information: Figure 1).

Lung cancers associated with cystic airspace have a higher risk of misdiagnosis due to lacking typical imaging characteristics of primary lung cancer. The diagnosis ability of F‐fluorodeoxyglucose positron emission tomography/computed tomography (F‐FDG PET/CT) is also limited for this type,^4^, ^5^ though the maximum standardized uptake value (SUV_max_) is higher in patients with small‐sized lung adenocarcinoma adjoining cystic airspaces.^6^ Previous studies showed that about 22% (5/22) of missed lung carcinomas originally presented as “bulla wall thickening” on CT,^7^ which suggested that timely detection of these lesions requires a better understanding of the early manifestation. However, little is known about the early indicators that a cystic airspace is a part of lung cancer but not a benign pulmonary cyst.^8^ Many studies attempted to find the characteristics of lung cancer associated with cystic airspaces^2^, ^9^, ^10^, ^11^: more males than females; with a mean age of 60–70 years; over 50% of patients have a smoking history; 80% of lesions were peripheral or subpleural and distributed in all lung lobes; most patients at stage I; about 80% was adenocarcinoma followed by squamous cell carcinoma; various molecular alterations with the most common are KRAS alteration and EGFR mutations.

Four patterns of lung cancer associated with cystic airspaces were recognized by Mascalchi et al.,^4^ according to the morphologic features of this lesion on CT scans. Type I and Type II refer to an exophytic or endophytic nodule or mass from the wall of cystic airspaces, respectively; Type III refers to cyst wall thickening without a focal nodule, which not necessarily be circumferential; and Type IV refers to solid or nonsolid nodule within a cluster of multicystic airspaces. A modified classification scheme including three aspects of airspace, consistency, and loculation was proposed by Fintelmann et al.,^2^ based on the work of Mascalchi et al.^4^ Mets et al.^12^ found that Type III is most often encountered compared with the other three types and the Type III and IV lesions may be more prone to misinterpretation compared to Types I and II which is with a solid component.

However, the current classification systems, which are morphologically divided according to the entity features of images on CT, neither represent the growth speed, biological behavior, and prognosis nor cover all lesion types. Moreover, one lesion may be divided into different types due to the different layers of CT images or interobserver variability.^12^ For this patient, the images of lung lesion presented as an eccentric thick‐walled cavity and exophytic nodules (Type I) or irregular wall thickening (Type III) from different layers in CT scans of three orthogonal axes in November 2020. Furthermore, different patterns may be transformed into each other with lesions progress,^4^ which also occurred in this case from a nonsolid nodule protruding externally from the cyst wall into a mixed type. So, three‐dimensional images and thin‐slice reconstructions of CT are needed for accurate classification and we should raise suspicion for cancer when the morphologic features of a cyst or pericystic nodule change. In addition, the cystic‐related lesion may change after antitumor therapy. Parisi et al.^13^ pointed out that the lesion types will change after chemotherapy and immune checkpoint inhibitor administration for advanced patients with cystic‐related lung cancer, which reflects treatment response.

Studies have indicated that the cystic airspaces developed wall thickening and/or a mural nodule with a median time of 25–35 months.^2^, ^4^ In fact, the evolution of a solid lung malignant lesion was consistently characterized by progressive wall thickening and/or the emergence of a nodule in or abutting the cyst wall.^14^ In this case, the time of a definitive progression from one purely thin‐walled lung cavity into lung malignant lesions was about 7 years, which reminds us a long follow‐up is necessary for the isolated emphysematous bullae.

There is no prospective data on prognosis and survival for patients with lung cancer associated with cystic airspaces. Results of an earlier study of lung cancers arising in bullous emphysema indicated that the overall prognosis was not substantially different from that of patients who had lung cancer not associated with bullae.^15^ However, a retrospective study showed that papillary (24/31) and solid (6/31) types are prevailing in patients with small‐sized lung adenocarcinoma adjoining cystic airspaces and these patients have poor disease‐free survival and overall survival.^6^ In this case, the lung adenocarcinoma lesion was with extensive papillary and micropapillary patterns. There is no tumor recurrence until April 2022. Recent studies have shown that three‐ITG signature (ITG subunit alpha 5 [ITGA5], ITG subunit alpha 6 [ITGA6], and ITG subunit alpha L [ITGAL]) could improve the prediction ability combined with pStage in lung adenocarcinoma and might contribute to poor prognosis by metastasis and immune escape‐related pathways.^16^

In conclusion, lung cancer associated with cystic airspaces is an uncommon manifestation of primary lung malignancy. More attention should be paid to the wall when lung cystic airspace is followed up by CT.

## AUTHOR CONTRIBUTIONS

Xue Yang wrote the manuscript. Linlin Zhang and Fanlu Meng provided patient information and collected the data. Dong Li and Diansheng Zhong were responsible for the study conception and design. Wenjing Song reviewed the pathological sections and took pathological photos. Linlin Zhang and Diansheng Zhong critically revised the manuscript for intellectual content. All authors contributed to the article and approved the submitted version.

## CONFLICT OF INTEREST

The authors declare no conflict of interest.

## ETHICS STATEMENT

Written informed consent was obtained from the individual for the publication of any potentially identifiable images or data included in this article.

## Supporting information


**Supporting Information**.

## Data Availability

The original contributions presented in the study are included in the article/supplementary material, further inquiries can be directed to the corresponding author/s.
