# Supplementary figures and images for: Lung adenocarcinoma associated with cystic airspaces
Source: Chronic Dis Transl Med. 2022 Nov 17;9(1):58–62. doi: 10.1002/cdt3.51 (PMC10011662; doi:10.1002/cdt3.51)

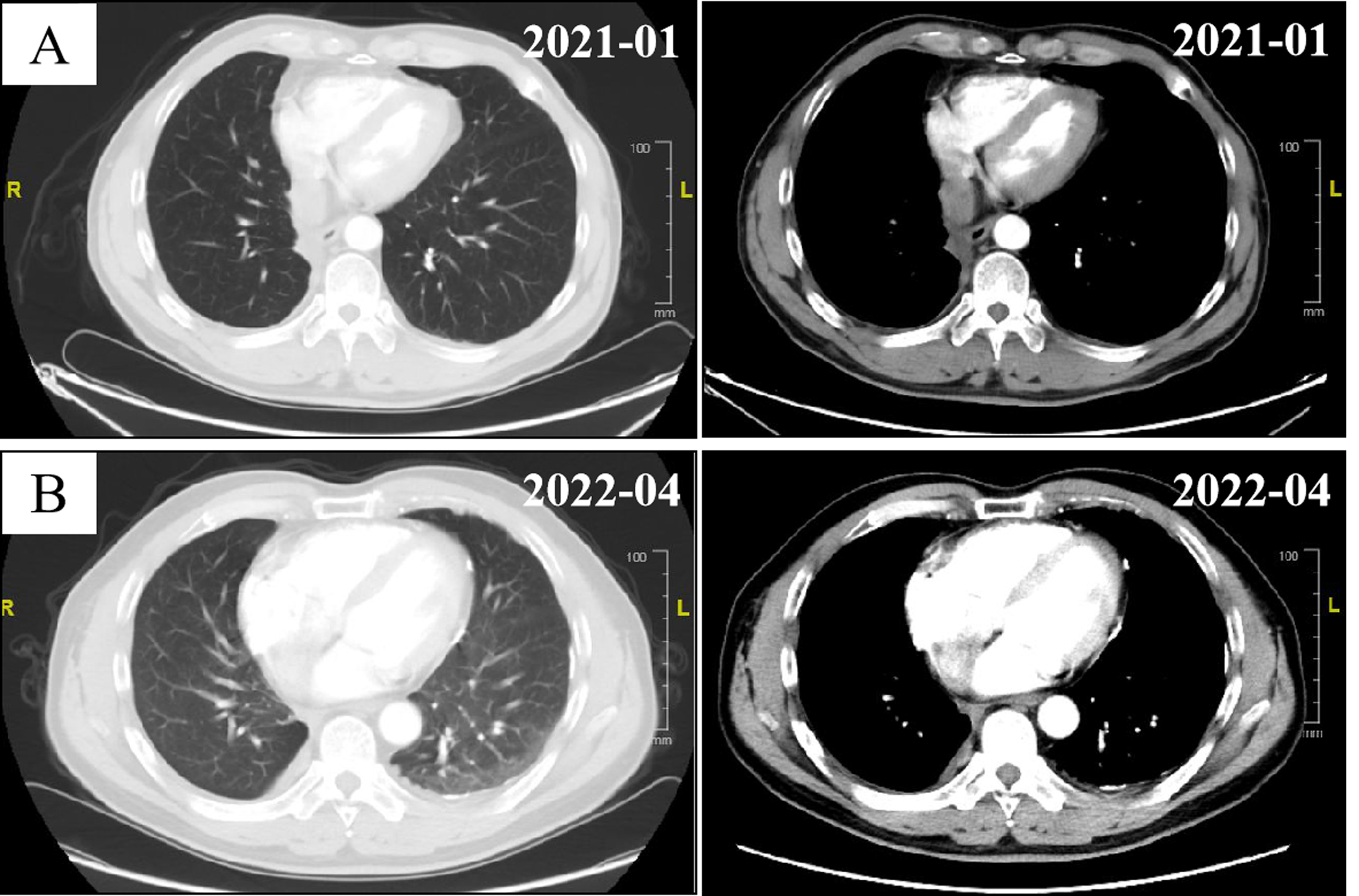

Supplement: Supplementary file 1 — Supporting Information. [file CDT3-9-58-s001.jpg]
